# CNS Involvement in a Patient with Chronic Myeloid Leukemia

**DOI:** 10.1155/2021/8891376

**Published:** 2021-03-11

**Authors:** Marcus A. Healey, Daniel J. Allendorf, Uma Borate, Ankit Madan

**Affiliations:** ^1^Edward Via College of Osteopathic Medicine, Blacksburg, VA, USA; ^2^Alabama Oncology, Alabaster, AL, USA; ^3^School of Medicine, Oregon Health & Science University, Portland, OR, USA; ^4^SOVAH Cancer Center, Danville, VA, USA

## Abstract

Inspite of medication compliance, some chronic myeloid leukemia (CML) patients will relapse/progress into an accelerated phase or blast crisis. Central nervous system (CNS) involvement is a rare manifestation of such a relapse. Here, we report a case of 23-year-old female who was diagnosed with CML in the accelerated phase and subsequently treated with imatinib. She developed early relapse in her CNS, and her treatment was switched to dasatinib and intrathecal chemotherapy with cytarabine and methotrexate. Her CNS disease went into remission, and she underwent matched unrelated donor (MUD) hematopoietic stem cell transplant (HSCT). We discuss various mechanisms of treatment failure, importance of vigilance for symptoms and signs of treatment failure/relapse, indications for use of different tyrosine kinase inhibitors (TKIs), and management of blast crises in CML.

## 1. Introduction

CML is a clonal proliferation of progenitor stem cells, resulting from a reciprocal translocation between chromosomes 9 and 22 forming the Philadelphia chromosome t (9; 22) (q34; q11). The chimeric BCR-ABL fusion gene resulting from this translocation subsequently codes for constitutively active tyrosine kinase that activates several intracellular proteins which leads to clonal proliferation. CML represents a success for targeted therapies known as tyrosine kinase inhibitors (TKIs). These agents are small molecule antagonists used in treatment of several cancers. In the case of CML, they are directed against the constitutively activated BCR-ABL protein. Approximately 90% of patients with CML are diagnosed in the chronic phase (CP). A fraction of patient's experience relapse or progress to an accelerated phase or blast crisis in the form of acute leukemia, either myeloid (AML) or lymphoid (ALL), respectively. Treatment with first generation TKI, imatinib, leads to complete hematologic, cytogenetic, and molecular remissions in approximately 90%, 70%, and 30% of patients, respectively [1]. The second generation TKIs, dasatinib and nilotinib, enjoy higher rates of cytogenetic and molecular remissions, roughly 90% and 70%, respectively, and these generally occur more rapidly than observed after treatment with imatinib [2, 3]. Patients require monitoring of complete blood count with differential every 2 weeks until patients attain a complete hematological response. Molecular response is measured by ratio of BCR-ABL1 transcripts to ABL1 transcript according to the international scale. This is performed by peripheral blood quantitative polymerase chain reaction (PCR). Cytogenetics should be performed in marrow cell metaphases at diagnosis through chromosome banding analysis or interphase fluorescence in situ hybridization (FISH), and in patients with atypical translocations, and when PCR is unable to measure atypical or rare BCR-ABL1 transcripts [4, 5]. These responses are measured against a set of temporal benchmarks that prompt changes in management if they are not met. It is reported that response rates for those patients presenting in the accelerated phase or blast crisis are decreased regardless of the TKI used but are still improved by the use of second generation TKIs [6]. Some patients fail to respond to treatment or relapse despite treatment. There are numerous mechanisms of treatment failures which include nonadherence, resistance, particularly in the form of ATP-binding pocket mutations within the BCR/ABL fusion protein, expression of transporter proteins, and epigenetic modifications of the fusion transcript, among others [7, 8]. Despite the achievement of therapeutic milestones including hematologic, cytogenetic, and molecular remissions, treatment failures as a consequence of above mechanisms can occur. Clinicians should always remain vigilant for evidence of treatment failures despite these milestones and change management appropriately. We report a patient undergoing treatment for CML with TKIs who had a very unusual presentation of relapsed disease, presenting with CNS involvement, despite adherence to therapy. We will discuss potential mechanisms of disease resistance or progression and briefly discuss the management of CML in blast crisis.

## 2. Case Presentation

Our patient is a 23-year-old female who presented with two weeks of fatigue, symmetric lower extremity edema, and easy bruising. Her white blood cell count (WBC) was 385,000 cells/*μ*L with basophilia and 15% blasts, platelet count (PC) of 1 × 10^6^ cells/*μ*L, and hemoglobin of 8.2 g/dL. Bone marrow aspiration and biopsy (BMBx) showed a hypercellular marrow (>95% cellularity) with 14% blasts, having an immature myeloid immunophenotype. Karyotype and FISH from the bone marrow aspirate were positive for t (9; 22) (q34; q11). She was diagnosed with CML in the accelerated phase defined by the WHO criterion of 15% circulating blasts [9] Treatment began with imatinib 400 mg twice daily. Within two weeks, her WBC count had normalized. After 10 weeks, she achieved a complete hematological response (CHR) with normal blood counts. At this time, she developed headache. She reported adherence to imatinib therapy. Her neurological exam revealed normal cranial nerve examination, normal deep tendon reflexes, and intact motor and sensory exam. She was evaluated and empirically treated with nonsteroidal anti-inflammatory drugs (NSAIDS) but found no relief. One week later, she underwent a gadolinium-contrasted MRI brain revealing enhancement in the left posterior frontal lobe involving the subarachnoid space. A lumbar puncture revealed an elevated opening pressure and increased cerebrospinal fluid (CSF) cell count of 468 cells/*μ*L with 51% blasts with the same immature myeloid immunophenotype. FISH from the CSF revealed evidence of t (9; 22) (q34; q11) in 200/200 cells analyzed. At the writing of this report, mutational status of CSF sample and CD 93 was not available. At this time, her CBC was normal, and she continued to be in complete hematological remission. BMBx showed no evidence of CML, suggesting an isolated CNS relapse of CML in blast crisis as per the WHO criterion of extramedullary infiltration of blasts [10]. Based on data suggesting superior CNS penetration by dasatinib, imatinib was discontinued, and dasatinib 100 mg daily was started [11–13]. In addition, she received one dose each of intrathecal cytarabine and intrathecal methotrexate on consecutive days. Thereafter, she received weekly intrathecal cytarabine for two additional weeks at which time the myeloid blast population was not detectable in the CSF. She continued to have no evidence of recurrent myeloid blasts in the CNS for six months after her relapse. Her CBC remained normal. BMBx performed four months after the relapse demonstrated no evidence of t (9; 22) (q34; q11) by karyotype or FISH, and quantitative reverse transcriptase PCR (QT-PCR) from the peripheral blood revealed no copies of the BCR/ABL transcript. Given her young age, outstanding performance status, the good support system, and the aggressive nature of her CML, she underwent a matched unrelated donor (MUD) hematopoietic stem cell transplant (HSCT) in the absence of a suitable sibling donor.

## 3. Discussion

Isolated CNS symptoms are rare in CML; however, there have been case reports that show a pattern of demographic data. Typically, but not seen in our case, males are more affected than females at a ratio of 4.5 : 1. The median age at diagnosis is 40 years old, and patients who had isolated CNS relapse were on imatinib therapy for a median of 2.4 years. Importantly, headache and vomiting are the most common presenting symptoms in isolated CNS relapse, and their presentation should prompt clinical suspicion of CML relapse. Papilledema remains the most common finding on fundoscopy, and leptomeningeal enhancement is the most common finding on imaging [14].

Durable remissions in patients with CML can be achieved with TKIs. A fraction of patients, though, will fail to respond or will have disease progression to the accelerated phase or blast crisis. At present, there are multiple sets of criteria in use to define the accelerated phase or blast crisis. In terms of prognosis, the most important factor appears to be blast count with the poorest prognosis found at a peripheral blood count of >30% blasts [15]. Myeloid immunophenotype and the use of TKI therapy prior to blast crisis from either the chronic phase or accelerated phase portends a worse prognosis [16]. There are multiple observed and hypothesized mechanisms of TKI failure including, but not limited to, nonadherence, resistance, particularly in the form of ATP-binding pocket mutations within the BCR/ABL fusion protein, expression of transporter proteins, and epigenetic modifications of the BCR/ABL fusion transcript [6, 7]. In the event of treatment failure, relapse, or progression to blast crisis, the management depends on the immunophenotype of the blasts.

Nonadherence defined by the Basel Assessment of Adherence Scale with immunosuppressive medication has been demonstrated in up to one-third of patients taking imatinib, and correlation studies have demonstrated an inverse relationship between nonadherence and the probability of achieving a complete cytogenetic response [17–19]. Mutations in the BCR/ABL protein are found in more than 50% of patients' refractory to imatinib [20]. Indications for mutation analysis include failure to achieve a hematologic response by three months, minimal cytogenetic response by six months, relapse (including increases in BCR/ABL transcript by QT-PCR), and presentation in or progression to the accelerated phase or blast crisis [7, 19]. Many of these mutations occur in the adenosine triphosphate- (ATP-) binding pocket of the protein, and one of these, the T315I mutation accounting for up to 15% of mutations, confers resistance to imatinib, dasatinib, and nilotinib [6]. Mutation status can be detected by RT-PCR with sequence analysis, and the sensitivity of various BCR/ABL mutants to available TKIs has been assessed in vitro to help guide treatment [21]. Newer generation TKIs are being designed to overcome this current limitation [22]. For instance, ponatinib is a 3rd generation TKI that is FDA approved for all CML patients harboring the T3151 mutation. Ponatinib should be considered in CML patients who have failed first and second-line therapies either through relapse or inability to tolerate side effects. The European Leukemia Network also recommends using ponatinib in patients who are resistant to second generation TKI and do not have a specific mutation [4]. One recent study showed that the identification of CD93 on CML cells may be important in identifying a population of tumor cells that are resistant to TKI therapy [23]. Thus, other therapies may need to be considered in patients expressing this marker. Transporter proteins and drug efflux pumps contribute to the resistance of tumors to many conventional and targeted therapies. Treatment of CML with TKIs is no exception. The P-glycoprotein efflux pump and ATP-binding cassette (ABC) transporter have been identified on primary CML isolates and have been shown to expel TKIs [24]. Indeed, it has been shown that imatinib concentration in the CNS is decreased by the P-glycoprotein pump at the blood-brain barrier [25]. Epigenetic regulation of BCR/ABL and other genes relevant to the survival of CML tumor cells is also described. There is a recent description of epigenetic silencing through hypermethylation of microRNA-203 whose targets include ABL1. Restoration of microRNA-203 expression through transfection with a vector containing microRNA-203 was shown to decrease the expression of the BCR/ABL transcript in a CML cell line. Epigenetic silencing also occurs through polycomb group (PcG) proteins, which are thought to play an important role in stem cell differentiation and hematopoiesis [26]. Last, sirtuin 1 is a histone deacetylase that is upregulated in CML cells positive for CD34. In studies of patients with CML treated with imatinib, SIRT1 expression was diminished but not entirely absent. Thus, direct sirtuin 1 inhibition may prove to be a potential novel therapy for CML patients [27].

Any treatment decision regarding management of CML in blast crisis should include consideration of allogeneic HSCT. The immunophenotype of the blasts should be assessed to determine if the cells are myeloid or lymphoid. Treatment should include a TKI with the specific choice dependent on prior therapy and mutation analysis (Table 1) [20]. Treatment with TKI only may be a sufficient bridge to allogeneic HSCT or it can be combined with conventional AML or ALL induction regimens for myeloid and lymphoid blast crises, respectively. Ponatinib has shown promise as potential additional therapy with allogeneic HSCT in patients with extramedullary blast crisis harboring the T3151 mutation [28]. In the case of our presented patient, she only had evidence of myeloid blasts in the CNS, which is an uncommon presentation. Her treatment plan was influenced by evidence that imatinib has low CNS penetration, whereas dasatinib, in animal models, had improved CNS penetration [12]. In addition, dasatinib has been shown to decrease microglial and astrocytic neuroinflammatory responses, which may have important implications for therapy in isolated CNS blast crises [29]. Local control in the CNS was achieved further by using cytarabine, a drug with activity against myeloid blasts [10, 30].

In summary, significant therapeutic advances in the treatment of CML have been made in the past fifteen years, most notably, by the development of TKIs. Despite these advances, a fraction of patients fail to respond optimally to treatment while others will relapse while receiving therapy. The mechanisms driving these clinical challenges include nonadherence, acquired mutations in the BCR/ABL fusion protein, the expression of TKI efflux systems by tumor cells, and failure of epigenetic silencing of the BCR/ABL transcript. Treatment decisions in these situations need to be highly individualized. In addition to the development of newer TKIs (such as radotinib in South Korea) and next generation TKIs (such as ponatinib), emphasis should be placed on the development of novel strategies to combat other mechanisms of treatment failure [4]. Clinicians must also remain vigilant for evidence of therapeutic failure or progressive/relapsed disease and be aware of unusual patterns of treatment failure as illustrated by our case.

## Figures and Tables

**Table 1:** European Leukemia Network' summary of the most appropriate alternative therapeutic options based on BCR-ABL KD mutation status [4].

T315I	Ponatinib
F317L/V/I/C, T315A	Nilotinib, bosutinib^#^, or ponatinib

V299L	Nilotinib or ponatinib

Y253H, E255V/K, F359V/I/C	Dasatinib, bosutinib^#^, or ponatinib

^#^Limited in vivo data suggest resistance to bosutinib in vivo in patients with E255K and, to a reduced extent, the E255V mutation.

## Data Availability

Data used to support this study/case report are publicly available. Patient data or information has been deidentified.
